# In situ management options to improve crucian carp (*Carassius carassius*, L.) and brown trout (*Salmo trutta*, L.) population status in Central Europe: A case study from the Czech Republic

**DOI:** 10.1002/ece3.9107

**Published:** 2022-07-11

**Authors:** Roman Lyach

**Affiliations:** ^1^ Institute for Evaluations and Social Analyses (INESAN) Prague The Czech Republic

**Keywords:** fish conservation, fish growth, fish recruitment, fish re‐introduction, freshwater management

## Abstract

The crucian carp (*Carassius carassius*, L.) and the brown trout (*Salmo trutta*, L.) are vanishing from freshwater ecosystems of central Europe. To conserve both species, tailor‐made conservation management of habitats and populations of both species was implemented and tested in the Czech Republic (central Europe). This management was adjusted to reflect the ecological needs of both species. This study aimed to describe the results of a tested in situ management and to analyze the population growth of brown trout and crucian carp under ideal conditions. An experiment was performed at 14 small gravel pit lakes. Seven of them were adjusted to fit the crucian carp habitat requirements while the other seven were treated as a control group. The same experiment was done on 14 smaller streams and with brown trout. The occurrence and growth of crucian carp and brown trout were surveyed over 2 years. A significantly faster growth of both crucian carp and brown trout was observed on the adjusted lakes and streams in comparison to the control group ones. Trout and carp prospered on small streams and gravel pit lakes (respectively) that were free of angling, fish stocking, pollution, piscivorous predators, and competition with hybridizing species like Prussian carp (*Carassius auratus*, L.) or hatchery‐reared brown trout.

## Graphical abstract

This study aimed to describe the results of the tested in‐situ management and to add a management recommendation for any future conservation actions regarding the brown trout and the crucian carp. We found that the conservation of both species is doable and can be successful if the conservation management is perfectly adjusted to the life traits of both species.

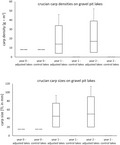

## 1 INTRODUCTION

We are currently experiencing a crisis of freshwater biodiversity. Most native wild fish populations in Europe have been declining in the last decades (years 1990–2022; Arlinghaus, 2006). This is true not only for commercially important fish species like brown trout (*Salmo trutta*, L.) but also for once very abundant fish species that were considered resilient to diverse threats (anthropogenic and natural) like crucian carp (*Carassius carassius*, L.; Copp & Sayer, 2020; Lusk et al., 2004; Mueller et al., 2018).

Both brown trout and crucian carp are important wild native species in central Europe, and a key part of the EU species conservation strategy (e.g., the Habitat Directive, the Water Framework Directive, and the EU Biodiversity Strategy for 2030). Brown trout is a key bioindicator species of water quality in small streams and a crucial vector of freshwater pearl mussel larvae in central Europe (Geist et al., 2006; Lusk et al., 2010; Simon et al., 2015). It is also an important prey item for the Eurasian otter *Lutra lutra* during cold winters when ponds and rivers freeze over (Lyach & Čech, 2017). Similarly, the crucian carp is an important inhabitant of periodically flooded ponds with oxygen deficiency (Copp et al., 2008; Holopainen et al., 1997; Nilsson et al., 1993; Poléo et al., 2017; Sayer et al., 2020; Sikorska et al., 2018; Tarkan et al., 2009). It also fills an important ecological niche as a food source for predatory fishes in sediment‐clogged ponds (Copp, 1989; Hänfling et al., 2005; Hohausová & Jurajda, 2005; Nilsson et al., 1993; Poléo et al., 2017; Wheeler, 1998).

However, both species are facing population declines due to numerous threats of anthropogenic origin (Almodóvar & Nicola, 2004; Copp et al., 2007, 2010; Dudgeon et al., 2006; Hao & Chen, 2008; Kottelat & Freyhof, 2007; Li et al., 2014; Reid et al., 2019). In central Europe, brown trout populations declined due to loss and degradation of small streams, inbreeding with hatchery‐reared nonlocal brown trout, competition with intensively stocked non‐native rainbow trout (*Oncorhynchus mykiss*, W.), high predation pressure from piscivorous birds and mammals, and due to intensive angling activities (Kranz, 2000; Lyach, 2020, 2022; Lyach et al., 2018; Musil et al., 2010). Other reasons for the population declines are lack of suitable spawning substrates due to sedimentation, intensive floods and droughts, fragmentation of streams, presence of stream obstacles, absence of underwater hideouts, and scarcity of insects as primary prey items (Almodóvar & Nicola, 2004; Hao & Chen, 2008; Louison & Stelzer, 2016; Machordom et al., 1999). The crucian carp populations further declined due loss and degradation of small ponds and cutoff rivers, limited food supply, and hybridization with its main competitor—the Prussian carp (*Carassius auratus*, L.; Hänfling et al., 2005; Sayer et al., 2011, 2013; Tarkan et al., 2011; Vetemaa et al., 2005; Wheeler, 2000; Wouters et al., 2012). However, since neither species is listed as endangered in the Czech Republic, a need for sustainable management of their populations has arisen.

While the anthropogenic threats to brown trout and crucian carp populations are generally known, there are not enough practical studies that would show successful mitigation of these threats in situ. Previous successful restoration projects of brown trout populations in western and northern Europe showed that trout needs naturally meandering and unpolluted streams without excessive angling pressure, stocking of non‐native fish (mainly rainbow trout *O. mykiss*), or otter predation pressure. The streams need to contain both deep parts (0.5 m) and shallow parts (with fast water) for hiding and spawning. These parts need to be connected to allow migration, meaning that any barrier higher than 0.5 m disallows smooth trout migration. A source of allochthonous insect prey needs to be available because trout feeds primarily on allochthonous insects (Louison & Stelzer, 2016; Machordom et al., 1999). Similarly, previous restoration projects of crucian carp populations in western and northern Europe showed that crucian carp prospers in small shallow ponds (under 2 m deep) without the presence of predatory fishes, anglers, otters, and—most importantly—invasive hybridizing Prussian carp (Copp et al., 2008; Holopainen et al., 1997; Nilsson et al., 1993; Poléo et al., 2017; Sayer et al., 2020; Sikorska et al., 2018; Tarkan et al., 2009).

As we can see above, successful projects that helped to preserve brown trout and crucian carp populations exist in western and northern Europe. However, to the best of our knowledge, there are no studies that would describe successful preservation projects of brown trout and crucian carp populations in central Europe (Matěna et al., 2017). Since the preservation strategy is always tailor‐made for local geographic conditions, this study will fill this knowledge gap by describing this in situ preservation management in central Europe.

In this study, an in situ experiment on crucian carp and brown trout populations was performed to test whether specific habitat adaptations corresponding to the species‐specific requirements can result in higher growth rates of local fish populations. We tried to copy the environmental conditions that were described in previously successful conservation projects, and we tried to apply them to central Europe. We wanted to see if brown trout and crucian carp would prosper in the newly created optimal conditions. It was hypothesized that the studied fish populations would grow faster and therefore prosper more in the adjusted habitats in comparison to the control group habitats.

## 2 METHODS

### 2.1 The study area

**FIGURE 1:**
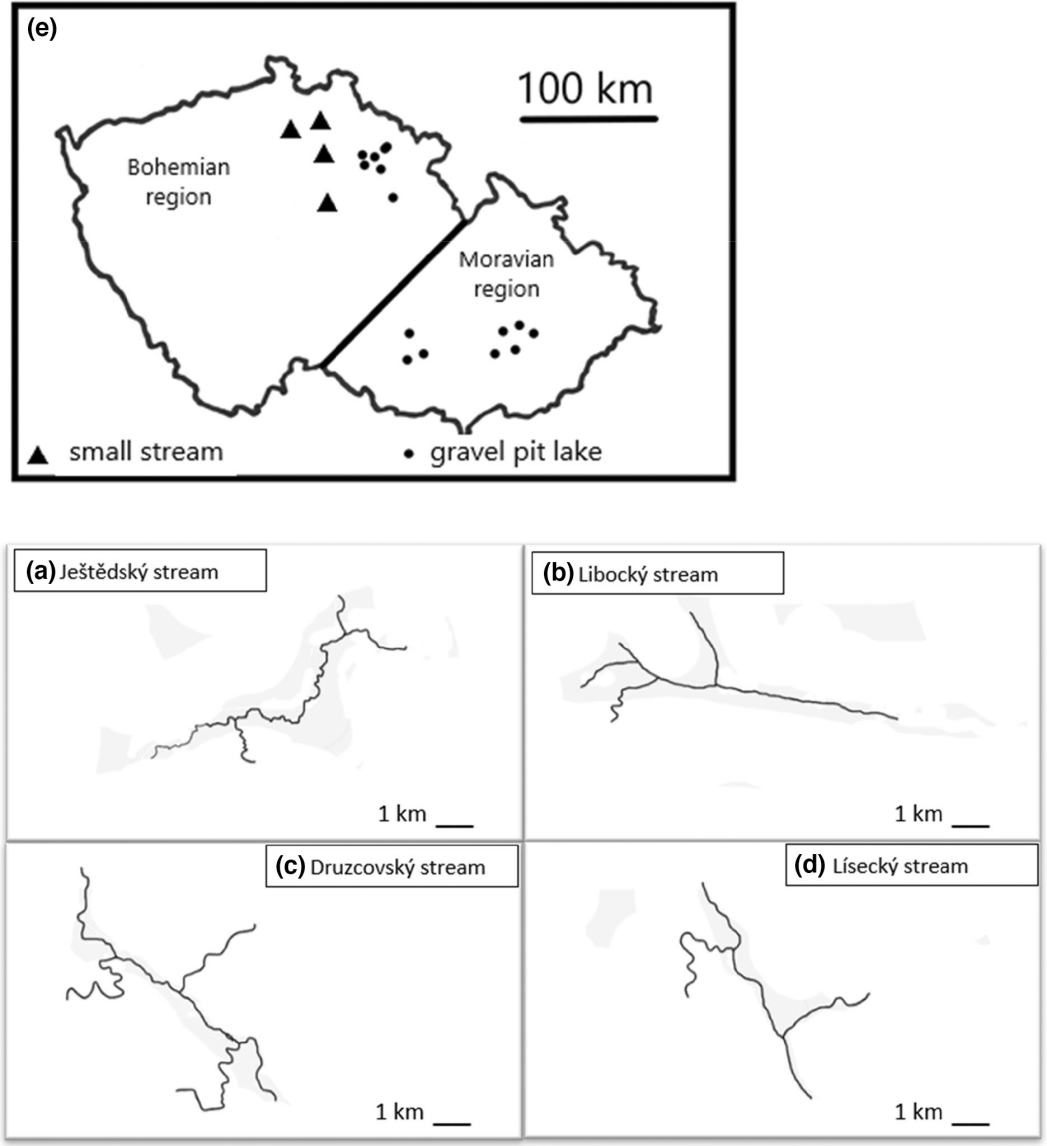
The map of the study area (the regions of Moravia and Bohemia in the Czech Republic) with highlighted streams and lakes where brown trout (*Salmo trutta*, L.) and crucian carps (*Carassius carassius*, L.; respectively) were sampled over years 2016–2018

The study was conducted on 14 smaller artificially made abandoned gravel pit lakes and on four smaller streams that had altogether 10 tributaries (Table S1a). This gives together 28 freshwater sampling locations (Figure 1). They were all situated in the eastern, northern, or central part of the Czech Republic (the Moravian region and Bohemian region) in central Europe (49.53 N, 17.07E—50.56 N, 15.93E). The streams belong to the Elbe River catchment (the North Sea drainage) while the pit lakes are in the Morava River catchment (the Black Sea drainage). The habitats are mesotrophic and located in lowlands (altitude 200–650 meters above sea level). The precipitation is between 200 and 400 mm annually and the temperatures are in the ranges of (−5)–(+5) and (+20)–(+30) degree Celsius during winter the summer, respectively.

### 2.2 Selection of test habitats

The experimental and control habitats were selected by the research team in cooperation with local fisheries managers and fish conservation experts from local branches of state environmental agencies (Table S2). We chose the study habitats based on our own experience with local brown trout and crucian carp populations in combination with the tips for proper habitats from scientific studies that describe successful trout and carp conservation projects (Copp et al., 2008; Holopainen et al., 1997; Louison & Stelzer, 2016; Machordom et al., 1999; Nilsson et al., 1993; Poléo et al., 2017; Sayer et al., 2020; Sikorska et al., 2018; Tarkan et al., 2009).

**FIGURE 2:**
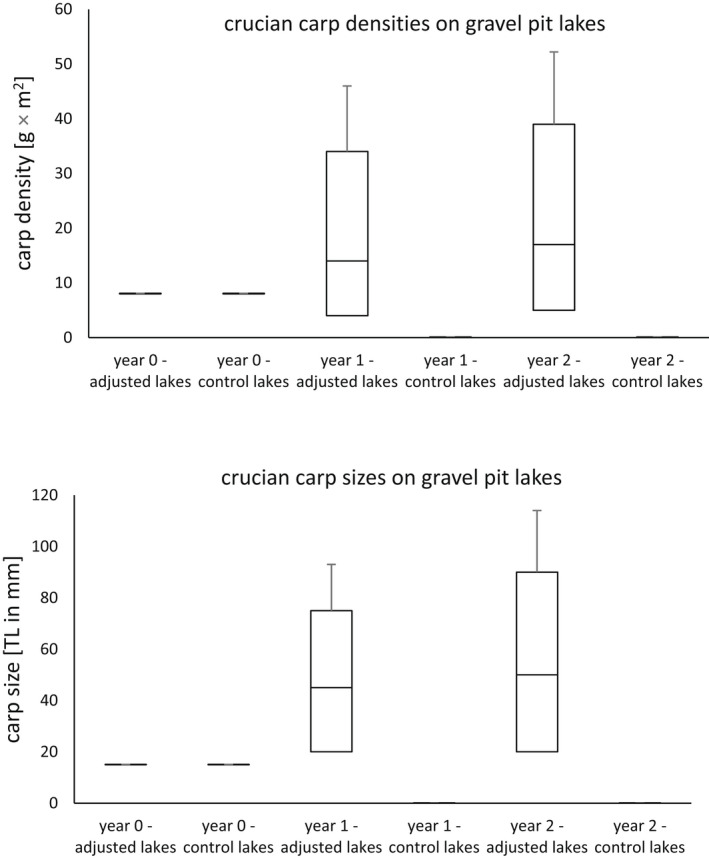
The population density (upper graph) and the body length (lower graph) of crucian carp (*Carassius carassius*, L.) in adjusted and control group lakes in the regions of Moravia and Bohemia (the Czech Republic) over years 2016–2018

**FIGURE 3:**
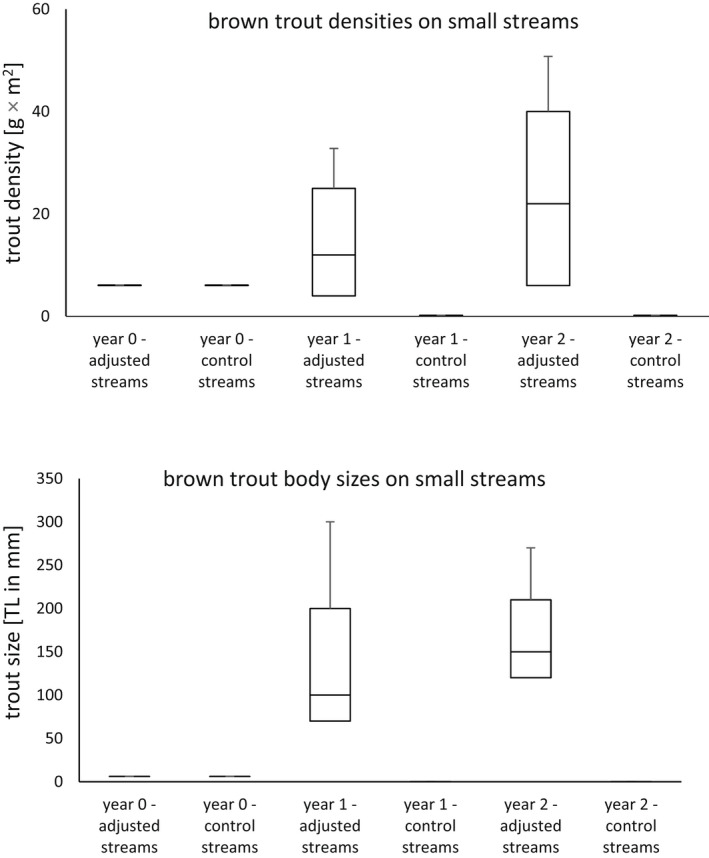
The population density (upper graph) and the body length (lower graph) of brown trout (*Salmo trutta*, L.) in adjusted and control group lakes in the regions of Moravia and Bohemia (the Czech Republic) over years 2016–2018

In the case of brown trout, we chose small streams (<4 m wide and at least 0.4 m deep) with natural meanders and shelters that allow trout migration and can sustain trout populations. It is because brown trout needs fast‐flowing highly oxygenated streams with the presence of shelters due to highly territorial behavior of adults. We chose streams with high oxygen levels (0.7–0.9 mg/L), lower temperature (10–13°C), and average pH and conductivity to ensure that trout does not suffocate or get intoxicated. The streams were located far (at least 100 m) from human settlements to avoid direct anthropogenic disturbances. It is because trout is vulnerable to disturbing and handling. The streams were also free of angling, fish stocking, and presence of Eurasian otter tracks. It is because trout is vulnerable to angling pressure and otter predation. We chose streams located in forest because trout needs a source of allochthonous prey, mainly insects (Figures 2 and 3).

In the case of crucian carp, we chose small and shallow gravel pit lakes (under 2 m deep with maximum of 150 m^2^ of water surface) because crucian carps require predator‐free waters, but they do not require a large water body. We chose lakes with sufficient oxygen levels (0.5–0.7 mg/L), suitable temperature (12–20°C), and average pH and conductivity to ensure that carps do not suffocate or get intoxicated. The lakes were located far (at least 100 m) from human settlements to avoid direct interaction with humans. It is because crucian carps are often taken as bait fish by anglers, and they suffer from stocking of competitive and predatory fishes. They also need to be free of otter tracks because crucian carp is an ideal prey item for otters due to inability to hide easily in smaller lakes. We chose lakes located in forest because such small lakes requite a source of allochthonous nutrients in the form of leaves and surface nutrient runoff.

### 2.3 Adjustment of test habitats

To conduct the experiment, the 28 studied locations were separated into two halves. One half of the habitats was an experimental one and the other was a control one (Table S1a). The experimental habitats were adjusted for the experiment (Table S2) using the techniques that are generally accepted to fit the ecological niche of the studied species and positively affect their populations (crucian carp: Copp et al., 2008; Holopainen et al., 1997; Nilsson et al., 1993; Poléo et al., 2017; Sayer et al., 2020; Sikorska et al., 2018; Tarkan et al., 2009; brown trout: Machordom et al., 1999; Louison & Stelzer, 2016). The remaining control habitats were left with no adjustments (Table S2).

The potentially suitable gravel pit lakes and small streams (Table S2) were first surveyed for an occurrence of crucian carp and brown trout (respectively) and for other fish species. A battery‐charged electrofishing device (LENA, 240 V, 60 Hz) was used to survey fish occurrences during March and April 2016 (Table 1). If at least five crucian carps or brown trout were found, the lake or stream was selected for this experiment. If predatory, competitive, or hybridizing fish species were detected in the lakes and streams where crucian carps and trout were introduced, the lake or stream was completely freed from all those fish and only the crucian carps and brown trout were returned to the lake (Beamesderfer, 2000). The remaining fish were transported and restocked in other pit lakes and streams closely located freshwater habitats. The fish were handled, transported, and released carefully to ensure their maximum wellfare through the entire operation.

**TABLE 1:** Timeline of fish surveys and habitat alterations on 14 studied small streams and 14 studied gravel pit lakes in the Czech Republic over years 2016–2018

Month	Year	Activity
3	2016	First (initial) surveying of small streams for brown trout
3	2016	First (initial) surveying of gravel pit lakes for crucian carp
4	2016	Alteration of small streams
4	2016	Alteration of gravel pit lakes and addition of crucian carp
4	2017	Second survey of growth of brown trout on small streams
4	2017	Second survey of growth of crucian carp on gravel pit lakes
4	2018	Third survey of growth of brown trout on small streams
4	2018	Third survey of growth of crucian carp on gravel pit lakes

Since crucian carp can easily be confused with other carp species (Ribeiro et al., 2015), all carps were carefully examined to match the species‐specific morphological traits. In addition, five randomly selected carps from the location were genetically examined by a DNA test in the laboratory using a small part of the scale (Baranov & Vasil'ev, 2018). If no crucian carps were detected but the lake was still evaluated as suitable habitat for carp population, the crucian carps were obtained from a local small fishpond owner and artificially stocked into the lake. The obtained carps were again confirmed using morphological traits and genetical examination of five randomly selected carps. The stocked fish were all larger than 90 mm and sexually mature to ensure that they can start reproducing for the purpose of the study. Similarly, the trout were examined and identified based on species‐specific morphological traits (Pakkasmaa & Piironen, 2001).

### 2.4 Surveying the growth and the population density of the studied carp and trout

The populations of crucian carp and brown trout were surveyed on the studied lakes and streams exactly 1 and 2 years (April 2017 and 2018), respectively, after the initial adjustments of the habitats (Table S1b,c). Each time the site was surveyed, the caught fish were measured, weighed, and released back to water. When the growth of the surveyed fish was calculated, only 1+ fish (the fish that were older than 1 year) were measured to avoid measuring the YOY (young of the year) offspring of the original population. We wanted to see if the original YOY fish from the first year grew, so we avoided measuring the newly hatched YOY fish. Similarly, in the second year, only 2+ fish were measured. The fish from the original population were distinguished from the newly hatched fish using a noninvasive scale analysis (Tarkan et al., 2016). When the population density of the surveyed fish was measured, all the fish (including YOY) were accounted for. We wanted to survey the whole population including the YOY fish to analyze the changes in population densities. The changes in the size were measured in TL (mm) while the changes in the population density were measured in (g) per (m^2^) of water surface.

In addition to surveying brown trout and crucian carp populations, we also tested whether environmental abiotic conditions (temperature, oxygen levels, pH, conductivity) have changed over time. We did this to see if population changes have a relationship with changes in environmental conditions.

### 2.5 Fish handling ethics

The fish were stocked and relocated with permission of the regional Angling Union (Český rybářský svaz—ČRS) branch and the Nature Conservation Agency of the Czech Republic (Agentura ochrany přírody a krajiny České republiky—AOPK ČR). Anglers, fisheries managers, and environmentalists who have the permission to handle live fish performed the experiment voluntarily and at their own expenses.

### 2.6 Statistical analysis

The statistical program R was used for the statistical testing (R Core Team, 2020). Firstly, Shapiro–Wilk test was used to analyze the distribution of the fish sizes and population densities. It was found that the data were not normally distributed (*p* < .01 in all the tests). Secondly, Lavene's test for homogeneity of variance was used on the tested parameters. It was found that there is a difference between the variances in the populations (*p* < .01 for all tests). Thirdly, the changes over time were tested within the environmental parameters to exclude the effect of environmental conditions on the growth of the fish and their population densities. No differences in environmental conditions were found between the tested years (Table S3).

For that reason, non‐parametric tests were used to analyze the fish sizes and population densities. Specifically, Wilcoxon test was used to compare the differences in the body sizes and population densities of the studied fish between the adjusted and the control habitats. When the body sizes and population densities of the studied fish were compared between years zero, one, and two, Kruskal–Wallis test (with a following Bonferroni correction test) was used to see if the fish sizes and fish densities changed. The alpha value of .05 was accepted in all the statistical tests, and all the tests were two‐tailed.

## 3 RESULTS

After the studied gravel pit lakes and small streams were adjusted to fit the ecological niche of crucian carp and brown trout (respectively), we observed that both species prospered significantly better on adjusted streams and lakes with optimal environmental conditions (Tables 2 and 3). Both crucian carp and brown trout had higher fish densities and faster body growths on the gravel pit lakes and small streams that were adjusted for their ecological niches in comparison to the lakes and streams that were not adjusted (the control group). When the growth was assessed on the adjusted lakes and streams between studied years (0, 1, and 2), it was discovered that the carps and trout significantly grew in both fish densities and body sizes (Tables 2 and 3). Conversely, no carps or trout were detected on the gravel pit lakes or small streams that were not adjusted to fit the ecological niche of the species. Therefore, no fish density or body size growth was observed on those lakes and streams. Since environmental conditions have not significantly changed over time (Table S3), we concluded that they did not significantly affect fish populations. The study hypothesis was confirmed, as the fish densities and growth rates were higher on the adjusted sites in comparison to the control ones.

**TABLE 2:** The differences in crucian carp densities and body sizes on the tested and control gravel pit lakes. The data were collected on 14 gravel pit lakes in the Czech Republic over years 2016–2018

Species	Tested parameter	Test result	*p*‐value	d.f.
Crucian carp	Difference in sizes of introduced and already existing fish on the tested lakes	Wilcoxon test = 8.42	<.01	
Crucian carp	Difference in population density growth on adjusted and control lakes in years 0 and 1	Wilcoxon test = 49	<.01	
Crucian carp	Difference in population density growth on adjusted and control lakes in years 1 and 2	Wilcoxon test = 52	<.01	
Crucian carp	Difference in fish size growth on adjusted and control lakes between years 0 and 1	Wilcoxon test = 10.75	<.01	
Crucian carp	Difference in fish size growth on adjusted and control lakes between years 1 and 2	Wilcoxon test = 9.75	<.01	
Crucian carp	Difference in fish sizes on adjusted lakes between years 0, 1, and 2	Kruskal–Wallis = 15.47	<.01	2

**TABLE 3:** The differences in brown trout fish densities and body sizes on tested and control sites (small streams). The data were collected on 14 small streams in the Czech Republic over years 2016–2018

Species	Tested parameter	Test result	*p*‐value	d.f.
Brown trout	Difference in sizes of introduced and already existing fish in the tested streams	Wilcoxon test = 5.37	<.01	
Brown trout	Difference in population density growth on adjusted and control streams in years 0 and 1	Wilcoxon test = 58	<.01	
Brown trout	Difference in population density growth on adjusted and control streams in years 1 and 2	Wilcoxon test = 43	<.01	
Brown trout	Difference in fish size growth on adjusted and control streams between years 0 and 1	Wilcoxon test = 21	<.01	
Brown trout	Difference in fish size growth on adjusted and control streams between years 1 and 2	Wilcoxon test = 35	<.01	
Brown trout	Difference in fish sizes on adjusted streams between years 0, 1, and 2	Kruskal–Wallis = 17.98	<.01	2

## 4 DISCUSSION

It was found that studied fish had higher fish densities and faster growth on the adjusted sites in comparison to the control sites. This confirmed the initial hypothesis that the carps and trout would prosper more on the adjusted sites.

The study managed to utilize the ability of the crucian carp to reproduce in suboptimal environmental conditions of a small gravel pit lake if they have no competition in the form of the invasive Prussian carp. This confirmed the findings of other authors who proved that crucian carp populations can be self‐sustainable if they have access to suitable habitats without a competition of invasive Prussian carp. The best strategy is to preserve small water bodies and to eradicate the Prussian carp populations (Sayer et al., 2020). Since crucian carp has a unique ability to survive in anaerobic conditions, it must be said that much more robust river restorations would be needed to save, for example, endangered rheophilic cyprinids (Holopainen et al., 1997; Wootton, 1998).

As far as the brown trout is concerned, the optimal abiotic conditions were more difficult to determine and adjust due to the trout's higher biological and physiological demands on environmental conditions (oxygen level, water clarity, temperature, spawning substrates etc.). There was a need to combine the improved flow permeability with creation of pools for overwintering and riffle‐pool segments for spawning. There was also the need to add barriers and hiding places to reduce the competition between the individual trout as well as to protect it from predatory otters and birds. Habitat alterations that other authors recommended (Baran et al., 1995; Matěna et al., 2017; Williams et al., 2006; Wootton, 1998) were used. The alterations included reduction of angling effort, fish stocking, and otter predation pressure. They also included removal of migratory barriers and installation of hiding places for trout. And trout populations prospered under these new conditions. Due to removal of migratory barriers, trout had access to deep waters and shelters to hide in and to shallow rapid waters for spawning. Absence of angling and stocking together with reduced otter presence further allowed trout populations to recover from predation pressure. Since environmental conditions did not change over the study period, the alterations probably led to growth of trout populations.

At the control sites, the populations either stagnated or declined, despite the habitats being selected as potentially suitable for these species (Table S2). Moreover, the studied crucian carps and brown trout were mostly observed in the control habitats during the first electrofishing survey, proving the occurrence of the species in the habitats. Unfortunately, when these control sites were sampled in the following years, there were few or no crucian carps or brown trout found. Perhaps, the control sites were chosen poorly despite having similar environmental parameters as the studied sites. It partially suggests that the crucian carp and brown trout populations are very prone to changing environmental conditions, and one extremely dry or cold year could potentially decimate them if the migration between the habitats is impossible or if there is no source population to supplement the sink population (Tarkan et al., 2011; Závorka et al., 2015). If we want to create a wild population of the crucian carp and the brown trout that is self‐sustainable, we must create suitable habitats, connect local populations, and allow for migration and replenishment (Hilderbrand & Kershner, 2000; Smialek et al., 2021).

Some of the adversary effects were out of hands of the managers—for example, if the water quality in the stream changed due to an emission of pharmaceutical or organic pollutants into the water, the trout population perished as well. It was because brown trout is susceptible to any kind of pollution (Durrant et al., 2011). Another reason why the promising trout populations vanished could have been an inappropriate artificial modification on the stream. For example, a potential straightening of the flow or construction of an upstream pond makes local droughts and floods more intensive which is suboptimal for local trout populations (Glińska‐Lewczuk & Burandt, 2011).

A big issue that could have affected the fish survival rates was the extreme drought in 2017 and 2018. This drought negatively affected most study areas that were surveyed in this research project. If the droughts return in the future, the crucian carp and brown trout populations will be in danger of perishing again. This drought could have been the main reason why fish populations perished on the control sites. Since climate change is connected to intensive droughts, the drought will likely return (Hänsel et al., 2019).

In addition, this project has several other caveats. The studied populations were observed and sampled for only 2 years and surveyed only 14 habitats per species in only one country in central Europe. Like in other studies, this limits the usage of the management advice to environmentally similar habitats only (Sayer et al., 2020; Tarkan et al., 2009). It is also likely that in 8–10 years or so, the invasive Prussian carp will invade the adjusted pit lakes. This could spell the end to all studied crucian carp populations. The obvious future goal is to prevent this from happening. However, since the populations were able to function in situ without any further management adjustments, it is a promising result.

There are other weaknesses as well. In the case of brown trout, fish growth and increased density could have been caused by downstream immigration of different trout from other streams (because the stream is an open system). Shifts in environmental conditions could have been responsible for the density shifts as well; even though the basic parameters (oxygen levels, temperature etc.) were all analyzed, there could be other factors that influenced the fish populations that we missed (Jonsson et al., 2001). Since trout and carps of different age classes (and sizes) grow at different speeds (younger fish grow faster than older ones), the faster growth on the studied sites could have been caused by overabundance of smaller fish.

On top of these weaknesses, we also do now know which adjustment of the streams and lakes had the strongest effect on the fish density and growth. It could have been the predator and competitor removal, the dredging, or the fish restocking. Any fish population is very likely to prosper when all predators and competitors are removed, so the effect of the adjustments is perhaps lower. It was also quite curious that the studied fish species mostly disappeared from control sites after 1 year. There is a possibility of some changes in biotic or abiotic factors intervening to our project that we missed.

Despite the weaknesses, there are several recommendations that can be drawn from the results of this study. In the case of crucian carp, small and shallow pit lakes (under 150 m^2^ and 2 m of depth) located in a forest proved to be suitable for carps. In the case of brown trout, small and shallow streams (less than 4 m wide and 0.6 m deep) located between forests and meadows proved suitable. The absence of anthropogenic activities, such as angling, pollution, or fish stocking also proved useful. The absence of piscivorous mammals and potentially hybridizing Prussian carps and hatchery‐reared brown trout was essential too.

## 5 CONCLUSION

This study showed us that the conservation of crucian carp and brown trout populations in situ is doable if both species have optimal conditions for their growth. Both species occurred much more frequently on the adjusted sites, suggesting the ability to perhaps survive there. If the managers use the advice that we gave in this paper, the preservation of crucian carp and brown trout to the nature of central Europe is achievable.

## AUTHOR CONTRIBUTIONS

**Roman Lyach:** Conceptualization (equal); data curation (equal); formal analysis (equal); funding acquisition (equal); investigation (equal); methodology (equal); project administration (equal).

## CONFLICT OF INTEREST

The author states that he has no competing interest.

## Supporting information


Table S1
Table S2
Table S3

## Data Availability

The data used to support the findings of this study are available publicly in an appendix and also on the Dryad repository: Lyach (2022), Brown trout and crucian carp population densities, Dryad, Dataset, https://doi.org/10.5061/dryad.9ghx3ffkw.
