# Supplementary material for: In situ management options to improve crucian carp (Carassius carassius, L.) and brown trout (Salmo trutta, L.) population status in Central Europe: A case study from the Czech Republic
Source: Ecol Evol. 2022 Jul 11;12(7):e9107. doi: 10.1002/ece3.9107 (PMC9273566; doi:10.1002/ece3.9107)
Supplement: Supplementary file 1 — Table S1 Table S2 Table S3 [file ECE3-12-e9107-s001.docx]

**Table S1 (A)** Description of morphological and environmental factors on the studied gravel pit lakes and small streams. The data were collected on 14 gravel pit lakes and 14 streams in the Czech Republic over years 2016–2018.

| location | habitat type | studied species | surface area [m^2^] | length [km] | depth [m] | oxygen levels [mg × l^-1^] | temperature of water [°C] | conductivity [mS × m] | pH |
| --- | --- | --- | --- | --- | --- | --- | --- | --- | --- |
| A | gravel pit lake | crucian carp | 100 | - | 1.2 | 0.4 | 13 | 49 | 6.8 |
| B | gravel pit lake | crucian carp | 95 | - | 1.4 | 0.5 | 15 | 30 | 7.1 |
| C | gravel pit lake | crucian carp | 104 | - | 1.1 | 0.5 | 12 | 30 | 7.2 |
| D | gravel pit lake | crucian carp | 95 | - | 1.1 | 0.6 | 16 | 57 | 7.5 |
| E | gravel pit lake | crucian carp | 88 | - | 1.9 | 0.5 | 18 | 38 | 8.1 |
| F | gravel pit lake | crucian carp | 103 | - | 1.9 | 0.7 | 12 | 48 | 7.9 |
| G | gravel pit lake | crucian carp | 76 | - | 1.7 | 0.6 | 15 | 45 | 7.7 |
| H | gravel pit lake | crucian carp | 112 | - | 1.1 | 0.5 | 14 | 56 | 7.7 |
| I | gravel pit lake | crucian carp | 86 | - | 1.3 | 0.6 | 16 | 57 | 7.8 |
| J | gravel pit lake | crucian carp | 60 | - | 1.4 | 0.5 | 17 | 53 | 7.6 |
| K | gravel pit lake | crucian carp | 109 | - | 1.5 | 0.6 | 15 | 39 | 7.6 |
| L | gravel pit lake | crucian carp | 86 | - | 1.4 | 0.7 | 16 | 34 | 7.9 |
| M | gravel pit lake | crucian carp | 103 | - | 1.5 | 0.5 | 15 | 56 | 7.5 |
| N | gravel pit lake | crucian carp | 108 | - | 1.8 | 0.5 | 20 | 56 | 7.5 |
| O | small stream | brown trout | 25 790 | 11 | 0.4 | 0.8 | 11 | 34 | 7.2 |
| P | small stream | brown trout | 26 550 | 10 | 0.5 | 0.9 | 10 | 25 | 7.2 |
| Q | small stream | brown trout | 29 740 | 12 | 0.5 | 0.8 | 12 | 41 | 7.4 |
| R | small stream | brown trout | 20 270 | 14 | 0.6 | 0.7 | 10 | 35 | 7.6 |
| S | small stream | brown trout | 1 803 | 2.5 | 0.6 | 0.8 | 10 | 45 | 7.7 |
| T | small stream | brown trout | 2 359 | 3.5 | 0.4 | 0.8 | 11 | 44 | 7.4 |
| U | small stream | brown trout | 1 997 | 2.5 | 0.4 | 0.9 | 10 | 29 | 7.2 |
| V | small stream | brown trout | 1 963 | 2.8 | 0.6 | 0.7 | 12 | 26 | 7.1 |
| W | small stream | brown trout | 2 928 | 3.6 | 0.5 | 0.9 | 13 | 29 | 7.7 |
| X | small stream | brown trout | 2 483 | 3.9 | 0.5 | 0.8 | 12 | 27 | 7.8 |
| Y | small stream | brown trout | 2 451 | 2.8 | 0.5 | 0.9 | 10 | 38 | 7.6 |
| Z | small stream | brown trout | 2 345 | 3.2 | 0.6 | 0.8 | 11 | 38 | 7.5 |
| ZA | small stream | brown trout | 2 357 | 3.1 | 0.5 | 0.9 | 12 | 39 | 7.4 |
| ZB | small stream | brown trout | 2 356 | 2.6 | 0.4 | 0.7 | 12 | 32 | 7.5 |

**Table S1 (B)** Description of the results of electrofishing surveys and fish stocking experiments on the studied gravel pit lakes and small streams. The data were collected on 14 gravel pit lakes and 14 streams in the Czech Republic over years 2016–2018.

| location | adjusted habitat | number of fish found using electrofishing | mean size of fish found using electrofishing [mm] | SD | number of fish stocked | initial fish density (wild + stocked) [g × m^2^] | first year fish density | second year fish density |
| --- | --- | --- | --- | --- | --- | --- | --- | --- |
| A | yes | 5 | 91 | 72 | 54 | 8 | 16 | 17 |
| B | yes | 10 | 99 | 45 | 48 | 8 | 20 | 22 |
| C | yes | 17 | 38 | 17 | 49 | 8 | 4 | 5 |
| D | yes | 12 | 17 | 8 | 51 | 8 | 2 | 2 |
| E | yes | 19 | 51 | 44 | 46 | 8 | 10 | 12 |
| F | yes | 0 | - | - | 46 | 8 | 30 | 32 |
| G | yes | 0 | - | - | 47 | 8 | 10 | 12 |
| H | no | 23 | 47 | 44 | 47 | 8 | 0 | 0 |
| I | no | 15 | 23 | 20 | 52 | 8 | 0 | 0 |
| J | no | 15 | 83 | 66 | 49 | 8 | 0 | 0 |
| K | no | 22 | 47 | 26 | 52 | 8 | 0 | 0 |
| L | no | 17 | 64 | 55 | 46 | 8 | 0 | 0 |
| M | no | 9 | 27 | 18 | 47 | 8 | 0 | 0 |
| N | no | 0 | 81 | 69 | 50 | 8 | 0 | 0 |
| O | yes | 19 | 127 | 101 | 102 | 6 | 15 | 25 |
| P | yes | 20 | 162 | 128 | 109 | 6 | 20 | 40 |
| Q | yes | 19 | 250 | 204 | 110 | 6 | 4 | 6 |
| R | yes | 6 | 99 | 81 | 96 | 6 | 8 | 6 |
| S | yes | 10 | 150 | 108 | 107 | 6 | 12 | 20 |
| T | yes | 19 | 225 | 213 | 102 | 6 | 25 | 35 |
| U | yes | 22 | 66 | 42 | 99 | 6 | 11 | 22 |
| V | no | 6 | 163 | 127 | 90 | 6 | 0 | 0 |
| W | no | 23 | 162 | 135 | 97 | 6 | 0 | 0 |
| X | no | 23 | 203 | 188 | 108 | 6 | 0 | 0 |
| Y | no | 18 | 158 | 134 | 103 | 6 | 0 | 0 |
| Z | no | 6 | 44 | 31 | 99 | 6 | 0 | 0 |
| ZA | no | 8 | 272 | 208 | 110 | 6 | 0 | 0 |
| ZB | no | 11 | 218 | 173 | 98 | 6 | 0 | 0 |

**Table S1 (C)** Description of the results of electrofishing surveys on the studied gravel pit lakes and small streams. The data were collected on 14 gravel pit lakes and 14 streams in the Czech Republic over years 2016–2018.

| location | adjusted habitat | initial fish size (wild + stocked) [mm] | first year fish size | SD | second year fish size | SD |
| --- | --- | --- | --- | --- | --- | --- |
| A | yes | 15 | 30 | 15 | 40 | 32 |
| B | yes | 15 | 35 | 21 | 45 | 38 |
| C | yes | 15 | 25 | 12 | 35 | 18 |
| D | yes | 15 | 20 | 13 | 30 | 23 |
| E | yes | 15 | 25 | 17 | 30 | 21 |
| F | yes | 15 | 20 | 13 | 20 | 16 |
| G | yes | 15 | 25 | 21 | 30 | 25 |
| H | no | 15 | - | - | - | - |
| I | no | 15 | - | - | - | - |
| J | no | 15 | - | - | - | - |
| K | no | 15 | - | - | - | - |
| L | no | 15 | - | - | - | - |
| M | no | 15 | - | - | - | - |
| N | no | 15 | - | - | - | - |
| O | yes | 5 | 70 | 55 | 120 | 101 |
| P | yes | 5 | 80 | 69 | 130 | 115 |
| Q | yes | 5 | 90 | 81 | 160 | 132 |
| R | yes | 5 | 100 | 77 | 160 | 134 |
| S | yes | 5 | 80 | 67 | 200 | 147 |
| T | yes | 5 | 100 | 78 | 200 | 153 |
| U | yes | 5 | 60 | 31 | 180 | 144 |
| V | no | 5 | - | - | - | - |
| W | no | 5 | - | - | - | - |
| X | no | 5 | - | - | - | - |
| Y | no | 5 | - | - | - | - |
| Z | no | 5 | - | - | ­- | - |
| ZA | no | 5 | - | - | - | - |
| ZB | no | 5 | - | - | - | - |

| **Table S2** Description of the surveyed environmental parameters and the management adjustments of the studied gravel pit lakes and small streams in the Czech Republic in 2016.   \| (A) crucian carp \| \| \| --- \| --- \| \| selection parameter of the pit lake \| adjustment of the pit lake \| \| small pit lake (max 150 m2 water surface) \| relocation of prussian carp \| \| shallow pit lake (under 2 m depth) \| relocation of competing and predatory fishes \| \| located far from human settlements and larger rivers (min 100 m) \| deepening the pit like to 1.5 m minimum \| \| no signs of otter tracks nearby \|  \| \| located in a forest (a source of allochthonous nutrients) \|  \| \| no connection to pollution sources \|  \| \| no angling or fish stocking is allowed in the pit lake \|  \| \|  \|  \| \|  \|  \| \| (B) brown trout \| \| \| selection parameter of the stream \| adjustment of the stream \| \| small stream (<4 m wide) \| relocation of competing and predatory fishes \| \| sufficient depth (minimum 0.4 m) \| removal of migratory barriers \| \| located far from human settlements and larger rivers (min 100 m) \| installation of plastic pipes (15 x 0.3 x 70 cm), one pipe per one km of the stream \| \| no angling or fish stocking is allowed on the stream \| installation of wooden planks (5 x 2 x 50 cm), one plank per one km of the stream \| \| located in a forest (a source of allochthonous prey items) \|  \| \| occurrence of brown trout in the stream \|  \| \| no signs of otter tracks nearby \|  \| |  |  |  |  |  |  |
| --- | --- | --- | --- | --- | --- | --- | --- | --- | --- | --- | --- | --- | --- | --- | --- | --- | --- | --- | --- | --- | --- | --- | --- | --- | --- | --- | --- | --- | --- | --- | --- | --- | --- | --- | --- | --- | --- | --- | --- | --- | --- | --- | --- | --- | --- | --- |

**Table S3** Results of the testing of changes within environmental parameters of the studied gravel pit lakes and small streams in the Czech Republic in 2016.

| habitat | parameter | test result | p-value | d. f. |
| --- | --- | --- | --- | --- |
| gravel pit lakes | oxygen levels [mg × l^-1^] | Kruskal-Wallis = 2.36 | 0.72 | 2 |
| small streams | oxygen levels [mg × l^-1^] | Kruskal-Wallis = 3.18 | 0.44 | 2 |
| gravel pit lakes | temperature of water [°C] | Kruskal-Wallis = 0.71 | 0.63 | 2 |
| small streams | temperature of water [°C] | Kruskal-Wallis = 1.12 | 0.39 | 2 |
| gravel pit lakes | conductivity [mS × m] | Kruskal-Wallis = 4.15 | 0.48 | 2 |
| small streams | conductivity [mS × m] | Kruskal-Wallis = 3.87 | 0.68 | 2 |
| gravel pit lakes | pH | Kruskal-Wallis = 4.41 | 0.79 | 2 |
| small streams | pH | Kruskal-Wallis = 3.57 | 0.12 | 2 |
